# Identification and Characterization of a Novel Bovine Adenovirus Which Represents a Distinct Evolutionary Branch

**DOI:** 10.3390/v18050522

**Published:** 2026-04-30

**Authors:** Jinyu Sui, Suchun Wang, Zihao Pan, Kaicheng Wang

**Affiliations:** 1 China Animal Health and Epidemiology Center, Qingdao 266032, China; suijinyu@cahec.cn (J.S.);; 2 Key Laboratory of Animal Biosafty Risk Prevention and Control (South), Ministry of Agriculture and Rural Affairs, Qingdao 266032, China; 3 Key Laboratory of Animal Biosafty Risk Prevention, Qingdao 266032, China; 4 College of Veterinary Medicine, Nanjing Agricultural University, Nanjing 210095, China

**Keywords:** bovine adenovirus, mastadenovirus, virus discovery, genome, novel species

## Abstract

Bovine adenovirus (BAdV) is associated with respiratory and enteric diseases in cattle. In this study, the complete genomic sequence of a novel BAdV strain (named BAdV/LN/CHN/2023) was sequenced and annotated using the next-generation sequencing (NGS) technology. The viral genome comprises 32,391 base pairs with a GC content of 44.93%, encoding 33 predicted open reading frames (ORFs), consistent with the genomic organization of mastadenoviruses. Comparative genomic analysis confirmed that BAdV/LN/CHN/2023 contains conserved structural and functional motifs characteristic of the genus Mastadenovirus. Phylogenetic analysis revealed that BAdV/LN/CHN/2023 shares low similarity with all currently recognized bovine mastadenoviruses classified by the International Committee on Taxonomy of Viruses (ICTV). In addition, an open reading frame (ORF) encoding the 146R protein was annotated in this strain; this feature has not been identified in any previously recognized bovine mastadenoviruses. This study presents the first full-length genomic sequence of a putative BAdV-11 strain, and based on ICTV criteria, we propose that this strain represents a novel mastadenovirus species, supported by phylogenetic distance and genomic divergence. Our findings expand the known genetic diversity of BAdVs and contribute to a better understanding of their evolutionary relationships.

## 1. Introduction

Adenoviruses (AdVs) are non-enveloped, linear double-stranded DNA viruses classified within the family Adenoviridae. These viruses are widely distributed across virtually all vertebrates, including mammals, birds, reptiles, amphibians, and fish, and numerous distinct viruses have been identified, with infections typically remaining subclinical, except for in young or immunocompromised individuals [1,2]. AdVs are remarkably stable in the environment, which potentially facilitates multiple modes of transmission [3]. According to the International Committee on Taxonomy of Viruses (ICTV), the family Adenoviridae is currently divided into six genera: Aviadenovirus, Barthadenovirus (formerly Atadenovirus), Ichtadenovirus, Mastadenovirus, Siadenovirus, and Testadenovirus [4]. Mastadenoviruses are capable of infecting a diverse range of mammalian hosts. Still, high host specificity is generally observed for these viruses, which typically infect only a single host species or evolutionarily closely related ones [5]. However, a few exceptions have been documented; for instance, human adenoviruses (HAdVs) can transmit bidirectionally between primates and humans [6,7]. Similarly, canine adenoviruses (CAdVs) are characterized by broad infectivity across multiple carnivore species [8,9].

Bovine adenovirus (BAdV) infection in cattle is highly prevalent across multiple countries worldwide, with a broad spectrum of clinical presentations ranging from asymptomatic to acute enteric disorders, respiratory syndromes, and weak calf syndrome in newborn calves [10,11,12]. Currently, BAdV-1~8, 10 have been described and classified into two genera, Mastadenovirus and Barthadenovirus. The genus Mastadenovirus encompasses BAdV-1 (species Bovine mastadenovirus A), BAdV-2 (species Ovine mastadenovirus A), BAdV-3 (species Bovine mastadenovirus B), and BAdV-10 (species Bovine mastadenovirus C). Members of the genus Barthadenovirus include BAdV-4, -5, and -8 (Bovine atadenovirus D), BAdV-6 (species Bovine atadenovirus E), and BAdV-7 (species Bovine atadenovirus F) [4]. In a diarrheal fecal specimen collected in Hungary, Europe, a study identified the following gene segments: hexon (GenBank accession: MK504013), DNA polymerase (pol) (GenBank accession: MK504014), IVa2 (GenBank accession: MK504014), and pVIII (GenBank accession: MK504015). The genomic entry MK504014 contains a 1503 bp fragment of the pol gene, corresponding to a partial coding sequence (CDS) that encodes 500 amino acids, and this encoded polypeptide shared 87% amino acid identity with the corresponding region of BAdV-2. Based on phylogenetic analysis of the partial pol sequence, the study proposed that the virus represents a novel adenovirus type and recommended its taxonomic classification as BAdV-11 [13]. However, owing to insufficient sequence coverage of the genomic regions critical for adenovirus species demarcation, the ICTV reported BAdV-11 on a list of viruses related to members of the genus Mastadenovirus, leaving the existence of a novel species unconfirmed. BAdVs adversely impact bovine health and welfare, increasing management and veterinary costs and posing significant health and economic challenges to the livestock industry [14]. BAdV-3 is a recognized pathogen implicated in bovine respiratory disease complex (BRDC) [15]. Additionally, BAdV-6, -7, and -10 are associated with respiratory or enteric clinical manifestations [11,16,17]. Pathogenicity, virulence, and tissue tropism vary among bovine adenovirus types, resulting in diverse clinical outcomes in infected cattle.

In this study, we report the identification and complete genome characterization of a novel BAdV, phylogenetically classified within the genus Mastadenovirus. Preliminary evidence suggests this isolate may be associated with clinical manifestations including bovine diarrhea, while its genomic characteristics suggest it may represent a distinct evolutionary lineage among mastadenoviruses.

## 2. Materials and Methods

### 2.1. Sample Collection

In October 2023, calves from a farm located in Liaoning Province, northeastern China, showed clinical signs of diarrhea. A fecal sample from one affected cow was collected fresh, transported on ice to the laboratory, and stored at −80 °C prior to further processing. The sample was homogenized with phosphate-buffered saline (1×PBS) and vortexed for 10 min, followed by three cycles of freeze-thawing. The homogenate was subsequently centrifuged at 12,000 rpm at 4 °C for 10 min. The resulting supernatant was sequentially filtered through 0.45 μm and 0.22 μm filters (Millipore) to eliminate bacterium-sized particles and eukaryotic cellular debris.

### 2.2. Library Preparation and Next-Generation Sequencing (NGS)

For the whole-genome and strain determination analysis, the QIAGEN Allprep PowerFecal DNA/RNA Kit (Qiagen, Hilden, Germany) was used to extract the total viral nucleic acids according to the manufacturer’s instructions. Library construction was performed using the Illumina DNA/RNA Preparation kit (Illumina, San Diego, CA, USA) and was uniquely tagged using a specific Illumina DNA/RNA UD index. Subsequent next-generation sequencing (NGS) was performed on an Illumina NextSeq^TM^1000 platform with paired-end reads (2 × 150 bp).

### 2.3. Genome Sequence Assembly and Annotation of Novel Virus

Quality trimming and de novo assembly were performed with CLC Genomics Workbench Version 26.0.1 (Qiagen, Aarhus, Denmark) with default parameters. A total of 14,096,732 clean reads were obtained. The assembled genome sequence was 31,839 bp in length, representing approximately 98.30% of the full genome (32,391 bp). To determine the terminal sequences of the genome, specific PCR primers were designed in this study based on the assembled contigs, and conventional PCR was used to amplify the corresponding fragments. Detailed information for these primers is provided in Supplementary Table S1. Genome annotation was performed utilizing the NCBI ORF Finder tool under the parameters of the standard genetic code, with ATG as the only start codon. Open reading frames (ORFs) were predicted with a minimum length of 300 nt, and overlapping ORFs were allowed. Predicted ORFs were subsequently verified and annotated through BLAST-based similarity searches against known viral genes in the GenBank database. No fixed thresholds for E-value, identity, or coverage were applied, and all significant hits were manually inspected and evaluated. The viral inverted terminal repeats (ITRs) were identified employing BLASTN (Basic Local Alignment Search Tool, https://blast.ncbi.nlm.nih.gov/Blast.cgi, accessed on 10 March 2026). The complete genome sequence of the BAdV (named “BAdV/LN/CHN/2023”) was submitted to the GenBank database with the accession number PX277336. The final viral genome architecture visualization was generated with SnapGene Viewer.

### 2.4. Phylogenetic Analysis and Recombination Analysis

Comparative genomic analysis of the sequence of this newly obtained virus was conducted against homologous sequences of representative members obtained from the GenBank database (Supplementary Table S2). Multiple sequence alignments and phylogenetic analyses were performed based on the amino acid sequence of pol and complete genomes using Molecular Evolutionary Genetics Analysis (MEGA) version 11.0 (Auckland, New Zealand). A maximum likelihood (ML) phylogenetic tree was constructed with bootstrap values based on 1000 replications. The amino acid substitution matrix model with gamma (LG + I + G) distribution was selected, and the robustness of the tree was tested using a non-parametric bootstrap calculation with 1000 replicates. Recombination between BAdV/LN/CHN/2023 and representative mastadenovirus reference strains was screened with Recombination Detection Program (RDP) version 4.100 (Imperial College London, London, UK) and Sequence Similarity Plotting (SimPlot) version 3.5.1 (Johns Hopkins University, Baltimore, MD, USA), using default parameter settings.

## 3. Results

### 3.1. Mastadenovirus Detection

Following high-throughput sequencing, a total of 15,204,254 reads were generated, and a complete BAdV genome was subsequently reconstructed by de novo assembly with PCR-based gap closure. This isolate, designated BAdV/LN/CHN/2023, has been deposited in GenBank under accession number PX277336. The final consensus genome comprises 32,391 base pairs (bp), with an average GC content of 44.93%, and the ITR is 174 bp. The genome sequence was confirmed to be complete through identification of the ITRs at both terminal ends of the genome, which serve as origins of viral DNA replication. BLASTn analysis against the NCBI nucleotide database revealed that the complete genome sequence of BAdV/LN/CHN/2023 shares 96.57% nucleotide identity with its closest known relative, Bovine adenovirus 11 strain (BAdV-11) (GenBank accession: MK504014), which has been identified in Belgian cattle. However, the alignment coverage between these two sequences is merely 0.07, due to the incomplete nature of the Belgian strain genome (2129 bp). The second closest relative strain is bovine adenovirus 2 (species Ovine mastadenovirus A, GenBank accession: NC_002513), with a genome size of 33,034 bp, sharing 74.33% nucleotide identity with limited query coverage of merely 16%.

### 3.2. Genome Characterization of Novel Mastadenovirus BAdV/LN/CHN/2023

**Figure 1:**
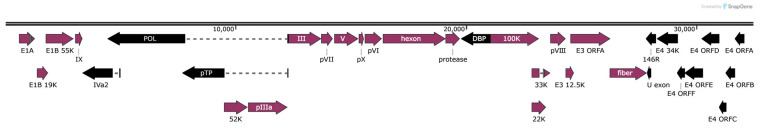
Gene map of BAdV/LN/CHN/2023.

The newly assembled genome contains 33 identified ORFs, all of which have been accurately annotated according to the ICTV description (Figure 1). The genome encodes genus-specific proteins V and IX, which are characteristic structural proteins of the genus Mastadenovirus, with the typical gene order of this genus [10,18]. Based on these characteristic features, BAdV/LN/CHN/2023 has been classified as a mastadenovirus. The identified ORFs of BAdV/LN/CHN/2023 were subjected to pairwise amino acid sequence alignment and subsequent comparative genomic analysis against corresponding sequences in the GenBank database. The majority of the identified ORFs (19/33) encode proteins that have significant sequence homology (25.88–85.26% amino acid identity based on BLAST analysis) to annotated gene products of bovine adenovirus A (BAdV-A) (GenBank accession: AC_000191), while nine ORFs encode proteins that share significant alignments with the annotated proteins from the ovine mastadenovirus A isolate (GenBank accession: AC_000001), with 56.00% to 88.11% identity to their closest BLAST homologs. Additionally, the highest similarity was observed for protein X relative to its counterpart in bat mastadenovirus WIV18 (GenBank accession: NC_035072), originally identified in Chinese fruit bats. Both protein pVI and fiber are most closely related to the corresponding gene products of porcine adenovirus 5 (GenBank accession: NC_002702), and protein hexon shared the closest homology with the caprine adenovirus 2 strain (GenBank accession: DQ630760) (Table 1). In conclusion, low overall similarity was observed between all coding regions of BAdV/LN/CHN/2023 and those of other mastadenoviruses.

### 3.3. Genomic Structural Divergence Between BAdV/LN/CHN/2023 and Other Bovine Mastadenoviruses

The overall genomic structure of BAdV/LN/CHN/2023 is generally comparable to that of BAdV-A (GenBank accession: AC_000191). A total of 33 putative ORFs are present in this strain, 32 of which are conserved in BAdV-A; however, amino acid sequence alignment reveals significant genetic differentiation between the two strains. Among these 33 ORFs, 19 correspond to annotated gene products of BAdV-A with significant sequence homology, although the overall amino acid identity is low, ranging from 25.88% to 85.26%. The divergence is particularly prominent in the E3 region: the amino acid identity of E3 ORFA is only 25.88% (Query Coverage: 56%), while that of E3 12.5K is 46.09% (Query Coverage: 96%), suggesting that BAdV/LN/CHN/2023 has undergone substantial genetic divergence in the E3 region.

Notably, a unique genomic characteristic is present in the E4 region of BAdV/LN/CHN/2023: an ORF encoding the 146R protein is located in this region. This ORF shows the highest sequence homology to the 146R protein of porcine adenovirus type 5, with 38.95% amino acid identity and 67% query coverage. To date, the 146R protein has not been identified in any other recognized member of the genus Bovine mastadenovirus, representing a unique feature of BAdV/LN/CHN/2023. These findings highlight the distinct genetic divergence of BAdV/LN/CHN/2023 from other bovine mastadenoviruses.

### 3.4. Phylogenetic Analysis and Whole-Genome Recombination Analysis

**Figure 2:**
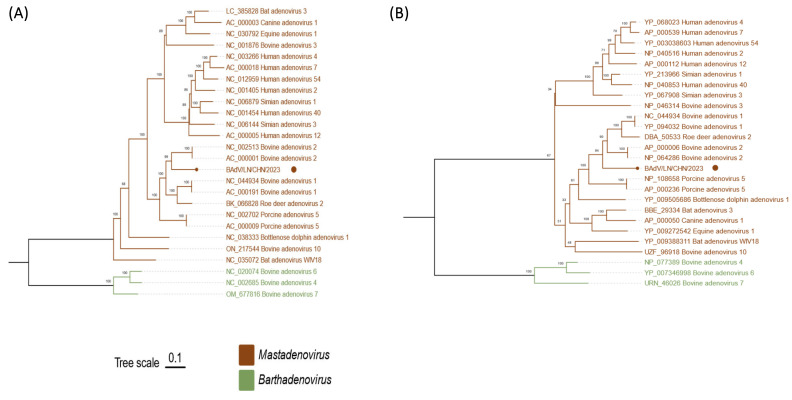
Phylogenetic analysis based on the whole-genome sequences (**A**) and complete amino acid sequences of pol (**B**). The bovine adenovirus identified in this study is indicated by a circle. Phylogenetic visualization and annotation were performed using ChiPlot (https://www.chiplot.online, accessed on 4 September 2025).

To determine the taxonomic identification of the mastadenovirus identified in this study, we conducted parallel maximum-likelihood (ML) phylogenetic reconstruction using the complete genome sequence (Figure 2A) and deduced pol amino acid sequence (Figure 2B), with the corresponding sequences of representative members of the genus Mastadenovirus and bovine viruses from the genus Barthadenovirus as outliers. The phylogenetic analysis based on the full-length genome revealed BAdV/LN/CHN/2023 as an evolutionarily distinct lineage, with nucleotide similarities ranging from 44.2% to 69.4% compared to other known MAdVs or BAdVs. Consistent with these findings, phylogenetic analysis of the deduced pol amino acid sequence supported its classification within a separate clade, with a minimum genetic distance of 26.2–29.7% from its closest relatives (BAdV-1 and BAdV-2), and significantly greater divergence from other adenoviruses (36.1–55.1%). No statistically significant recombination signals were detected, indicating that this strain may represent a distinct, independently evolving adenoviral lineage. Nevertheless, because recombination detection is dependent on the breadth of reference sequences, the absence of detectable breakpoints cannot formally exclude the possibility that an as-yet-unsequenced or unpublished adenovirus served as a parental donor in a recombination event that generated BAdV/LN/CHN/2023. These observations call for expanded epidemiological surveillance to clarify the evolutionary origin of this strain.

## 4. Discussion

Using NGS, we identified a novel bovine mastadenovirus, designated BAdV/LN/CHN/2023, and successfully determined its complete genome sequence. The mastadenovirus genomes vary between 27,952 (polar bear adenovirus 1) and 38,073 bp (bat adenovirus WIV11, Mastadenovirus rhinolopidae) [4,10,19] and nucleotide composition (GC) ranges between 31.3% (bat adenovirus 8 strain WIV13, Mastadenovirus humile) and 70.0% G + C (ovine adenovirus 8, Mastadenovirus octavum) [20]. The ITRs of mastadenoviruses are generally longer, ranging from 35 to 419 bp (bat adenovirus 4 strain WIV9 and bovine adenovirus 1) [4,19]. The genome size, GC content, and ITR length of the novel mastadenovirus are within the known range.

To date, more than 60 adenovirus species in the genus Mastadenovirus have been described [4]. Adenoviruses were traditionally classified serologically (by virus neutralization). However, the serological type demarcation criterion is currently being replaced by genomic criteria. According to the ICTV species delimitation criteria for the genus Mastadenoviruses, a total of nine criteria are provided, and fulfilment of at least two is required for designation as a new species. The phylogenetic distance (>10–15%, based on maximum likelihood analysis of the pol amino acid sequence) has been formally designated by the ICTV as the primary genomic criterion for species demarcation of mastadenoviruses, and a second widely recognized criterion is genome organization, particularly in the E3 region [4]. Based on the phylogenetic analysis of the pol amino acid sequences, significant differences (p-distance > 25%) were observed between BAdV/LN/CHN/2023 and established mastadenovirus references. Importantly, beyond the significant phylogenetic divergence of the pol amino acid sequence, unique genomic features further support that BAdV/LN/CHN/2023 represents a novel species within the genus Mastadenovirus. Although the overall genomic structure is generally similar to that of representative bovine adenoviruses, remarkable divergence was observed in the E3 and E4 regions. In the E3 region, the amino acid identities of major ORFs were extremely low (25.88% and 46.09%), indicating extensive genetic differentiation. Notably, a unique ORF encoding a 146R protein was identified in the E4 region, which has not been documented in any previously established bovine mastadenovirus species. Collectively, the distinct genomic characteristics, combined with the phylogenetic evidence, support the classification of BAdV/LN/CHN/2023 as a novel species within the genus Mastadenovirus.

Genomic comparison analysis indicated a high degree of sequence identity between BAdV/LN/CHN/2023 and the BAdV-11 strain previously identified in Hungary (GenBank accessions: MK504013, MK504014 and MK504015). Specifically, 96.74% nucleotide identity and 98.00% amino acid identity were observed for the pol gene. Similarly, the hexon gene was characterized by a near-perfect sequence match with 99.60% nucleotide identity and 100% amino acid identity, despite limited query coverage (9%). This suggests that BAdV/LN/CHN/2023 and the BAdV-11 strain may belong to the same species. Although the ICTV has not formally classified BAdV-11 as a novel species, our study successfully obtained its complete genome sequences, thereby providing critical evidence to support its classification as a novel BAdV species. The new BAdV-11 has been identified in geographically distant regions, with confirmed cases in Hungary (Europe) and China (Asia). This emerging BAdV appears to be persistently maintained within bovine populations and its present distribution likely reflects gradual geographic diffusion, potentially facilitated by animal movements or environmental factors. Notably, its true epidemiological prevalence may substantially exceed currently reported cases. However, differences in surveillance capabilities and other factors likely result in undetected occurrences of the pathogen in other parts of the Eurasian continent. Such surveillance bias leads to knowledge gaps regarding the true prevalence and transmission dynamics of this emerging pathogen on the continent. It is also possible that a similar evolutionary trend occurs in BAdVs, potentially driven by selection pressures such as host immune adaptation or environmental constraints.

A higher BAdV prevalence of 0.38 was observed in cattle herds affected by BRDC, further supporting the significant involvement of BAdV in BRDC [14]. However, the geographic distribution of BAdV and its pathogenic mechanisms, particularly its multifaceted impacts on BRDC, remain inadequately documented. Although BAdV-3 has been identified as a significant factor in BRDC [15], the roles of other types require further systematic research. Only this novel bovine adenovirus was identified by NGS, with no sequences of other known diarrhea-associated pathogens detected, including bovine rotavirus, bovine coronavirus, bovine viral diarrhea virus, or Cryptosporidium. However, targeted laboratory testing for these enteric co-infections was not performed, which represents a limitation of this study. Furthermore, viral isolation was not successful in this study, which precluded direct experimental confirmation of its pathogenicity. Therefore, the specific pathogenic role of BAdV/LN/CHN/2023 in the observed diarrhea cases remains unclear. Additionally, significant uncertainties persist regarding the molecular epidemiology and pathogenesis of BAdV-11, which is currently largely uncharacterized. We strongly recommend implementing systematic molecular surveillance programs and further viral isolation for BAdV-11 to accurately assess its distribution, clinical impacts, and independent pathogenicity in bovine production systems.

## 5. Conclusions

In conclusion, we have successfully determined the complete genome sequence of a novel bovine mastadenovirus. Based on phylogenomic and comparative genomic analyses, we propose that this strain be classified as a new species within the genus Mastadenovirus. The emergence of a novel bovine mastadenovirus in China signifies a notable shift in the circulating BAdVs within the country. The complete genomic data will serve as a valuable tool for in-depth investigation of pathogenic mechanisms and epidemiological characteristics.

## Figures and Tables

**Table 1:** Characterization of all the newly predicted genes in the BAdV/LN/CHN/2023 (PX277336) and their closest genes from the NCBI database.

	Gene Name	Gene Coordinates; Encoding Protein Length	Transcription Class	Nearest Homolog (Proteins)			
				Genbank ID	Query Coverage	Identity	Scientific Name
	*ITR*	1–174; 31,971–32,044					
1	*E1A*	355–796, 869–1032; 201 aa	E1A	YP_094027	99%	54.00%	Bovine Mastadenovirus A
2	*E1B 19K*	1123–1602; 159 aa	E1B	YP_094028	100%	57.23%	Bovine Mastadenovirus A
3	*E1B 55K*	1500–2717; 405 aa	E1B	YP_094029	100%	58.68%	Bovine Mastadenovirus A
4	*DBP*	19,539–20,813; 424 aa	E2A	YP_094041	100%	63.45%	Bovine Mastadenovirus A
5	*POL*	4194–7577, 12,014–12,022; 1130 aa	E2B	AP_000006	100%	71.83%	Ovine Mastadenovirus A
6	*pTP*	7424–9274, 12,014–12,022; 619 aa	E2B	YP_094033	100%	75.84%	Bovine Mastadenovirus A
7	*E3 ORFA*	24,301–26,022; 573 aa	E3	YP_094047	56%	25.88%	Bovine Mastadenovirus A
8	*E3 12.5K*	24,078–24,440; 120 aa	E3	YP_094046	96%	46.09%	Bovine Mastadenovirus A
9	*U exon*	27,621–27,785; 54 aa	E4	XII38533.1	98%	46.43%	Ovine Mastadenovirus A
10	*146R*	27,822–28,247; 141 aa	E4	NP_108676	67%	38.95%	Porcine adenovirus 5
11	*E4 34K*	28,046–28,936; 296 aa	E4	YP_094050	100%	49.83%	Bovine Mastadenovirus A
12	*E4 ORFD*	29,984–30,745; 253 aa	E4	AP_000020	100%	62.45%	Ovine Mastadenovirus A
13	*E4 ORFE*	29,266–30,024; 252 aa	E4	YP_094052	85%	50.23%	Bovine Mastadenovirus A
14	*E4 ORFA*	31,425–31,838; 137 aa	E4	YP_094055	92%	61.11%	Bovine Mastadenovirus A
15	*E4 ORFB*	31,033–31,428; 131 aa	E4	AP_000260	91%	33.61%	Ovine Mastadenovirus A
16	*E4 ORFF*	28,933–29,265; 110 aa	E4	YP_094051	98%	48.15%	Bovine Mastadenovirus A
17	*E4 ORFC*	30,738–31,052; 104 aa	E4	YP_094054	97%	43.52%	Bovine Mastadenovirus A
18	*52K*	9258–10,277; 339 aa	L1_1	YP_094034	94%	73.83%	Bovine Mastadenovirus A
19	*pIIIa*	10,304–11,998; 564 aa	L1_2	YP_094035	99%	71.23%	Bovine Mastadenovirus A
20	*III*	12,046–13,467; 473 aa	L2-1	YP_094036	98%	85.26%	Bovine Mastadenovirus A
21	*pVII*	13,469–13,975; 168 aa	L2-2	AP_000010	100%	67.80%	Ovine Mastadenovirus A
22	*V*	14,052–15,083; 343 aa	L2-3	AP_000011	100%	61.10%	Ovine Mastadenovirus A
23	*pX*	15,118–15,315; 65 aa	L2-4	YP_009388318	97%	65.08%	Bat mastadenovirus WIV18
24	*pVI*	15,380–16,102; 240 aa	L3-1	NP_108666	100%	55.87%	Porcine adenovirus 5
25	*hexon*	16,140–18,866; 908 aa	L3-2	DQ630760	100%	88.33%	Caprine adenovirus 2
26	*protease*	18,870–19,484; 204 aa	L3-3	YP_094040	100%	73.04%	Bovine Mastadenovirus A
27	*100k*	20,825–22,936; 703 aa	L4_1	AP_000015	98%	72.78%	Ovine Mastadenovirus A
28	*22K*	22,626–23,225; 199 aa	L4_2	AP_000017	66%	56.00%	Ovine Mastadenovirus A
29	*33K*	22,626–22,962, 23,135–23,424; 208 aa	L4	AP_000016	66%	61.84%	Ovine Mastadenovirus A
30	*pVIII*	23,417–24,085; 222 aa	L4_3	YP_094045	100%	73.09%	Bovine Mastadenovirus A
31	*fiber*	26,012–27,613; 533 aa	L5	NP_108675	98%	40.68%	Porcine adenovirus 5
32	*IX*	2784–3089; 101 aa	Intermediate	YP_094030	100%	51.30%	Bovine Mastadenovirus A
33	*IVa2*	3097–4430, 4709–4721; 448 aa	Intermediate	YP_094031	99%	72.97%	Bovine Mastadenovirus A

## Data Availability

The data presented in this study are available in the article. The novel adenovirus genome sequence generated in this study has been deposited in the National Center for Biotechnology Information (NCBI) GenBank under the accession number: PX277336.

## Supplementary Materials

The following supporting information can be downloaded at: https://www.mdpi.com/article/10.3390/v18050522/s1, Information on the adenovirus reference sequences used for analysis in this study is shown in Supplementary Table S1, Information of PCR primers designed in this study for genomic gap filling and terminal region amplification; Table S2, Reference adenovius used for analysis in this study.
